# Mechanisms underlying the biological changes induced by isocitrate dehydrogenase-1 mutation in glioma cells

**DOI:** 10.3892/ol.2014.1806

**Published:** 2014-01-16

**Authors:** JU-BO WANG, DAN-FENG DONG, KE GAO, MAO-DE WANG

**Affiliations:** 1 Department of Neurosurgery, First Affiliated Hospital of the Medical College of Xi’an Jiaotong University, Xi’an, Shaanxi 710061, P.R. China; 2 Department of Oncology, First Affiliated Hospital of the Medical College of Xi’an Jiaotong University, Xi’an, Shaanxi 710061, P.R. China

**Keywords:** isocitrate dehydrogenase 1, cell cycle, migration, invasion, matrix metalloproteinases

## Abstract

Isocitrate dehydrogenase 1 (IDH1) mutation has been reported to be associated with an increased overall survival in patients with glioma in a number of studies. Previous studies have focused on the mutation rate and possible metabolic pathways of the mutated IDH1 gene. However, the effects of IDH1 mutation on the biological behavior of glioma cells and the associated mechanisms, as well as the possible effects they may have on clinical therapy, have not been studied. In the present study, three eukaryotic expression vectors were constructed and transfected into the U87 cell line, specifically, a wild-type form of the IDH1 gene with the enhanced green fluorescent protein (EGFP) gene, a mutated IDH1 gene with the EGFP gene and the EGFP gene only. The three stable cell lines were selected using the G418 antibiotic. The biological behaviors of the cell lines were studied and the mechanisms underlying the biological differences between the cell lines were further investigated. The present study confirmed that IDH1 mutation induced cell cycle arrest in the G1 phase and reduced the proportion of the G2/M phase, by downregulating cell division control protein 2 homolog levels, increasing bromodomain-containing protein 2 levels and markedly limiting cell proliferation. IDH1 mutation had no effect on the apoptosis rate under routine culture conditions. Serum chemotaxis assays showed that IDH1 mutation was markedly associated with a significantly reduced invasion ability, by reducing the levels of matrix metalloproteinase (MMP)-2 and MMP-9. From this study, it may be concluded that IDH1 mutation improves prognosis in glioma patients by altering the cell cycle, inhibiting cell proliferation and downregulating cell invasion ability. The results may provide a partial explanation for the improved prognosis of patients with mutated forms of the IDH1 gene.

## Introduction

Gliomas are the most common and aggressive type of brain tumor. They account for 32–45% of all primary brain tumors (1,2) and 70–80% of all malignant brain tumors (2,3). A high disease incidence, mortality rate and disability rate place gliomas among the most fatal tumors. For the last two decades, important advances have been made in neurological and clinical surgery techniques. Gross total tumor removal, chemotherapy and radiotherapy have become standard treatments for newly diagnosed patients (4). Despite the most extensive treatment protocols, including clinical surgery, radiation treatment and chemotherapy, patients have a median survival time of only 12–15 months (5). This is due to the malignant behavior of the tumor and resistance to current therapeutic approaches (6). Patients usually undergo another surgery following a short period of remission. Additionally, a large number of patients succumb to glioma prior to a second surgery. More effective individualized treatment has become the focus of research on the treatment of glioma. Parsons *et al* presented researchers with a new direction for the study of glioma treatment following the observation that mutation of the isocitrate dehydrogenase 1 (IDH1) gene is frequent in glioma (7). IDH1 mutation may represent a new gene subtype of glioma and be an effective target for tumor therapy. IDH plays an important role in the tricarboxylic acid (TCA) cycle. Through evolution, humans have developed three IDH enzymes: NADP-dependent enzymes IDH1 and IDH2, and NAD-dependent enzyme IDH3. IDH1 is present in the cytoplasm and peroxisomes while IDH2 and IDH3 are found in the mitochondria. NAD^+^-specific IDH catalyzes a rate-limiting step in the TCA cycle. The affinity of yeast IDH for isocitrate is enhanced by AMP and reduced by NADH (8,9). The enzyme IDH1 catalyzes the citric acid oxidation of grass succinic acid and the subsequent oxidative decarboxylation generates α-ketoglutarate and produces NADPH (10). IDH enzymes are of great importance in the generation of biological energy and synthesis of metabolic pathways. Mutated IDH1 consumes rather than produces NADPH (10), thus markedly reducing NADPH levels. An increasing number of studies have shown that patients carrying mutated IDH1 genes have an improved prognosis. However, the mechanism by which the mutated IDH1 gene improves prognosis remains unclear.

In the present study, three cell lines stably expressing wild-type IDH1 (wIDH1), mutated IDH1 (mIDH1) and enhanced green fluorescent protein (EGFP) were constructed for the study of their effects on the biological behavior of glioma cells. The results aim to elucidate the mechanisms underlying these effects and provide clinicians with an overview of the current understanding of IDH1 mutation at the molecular level. This understanding is likely to lead to new therapeutic targets and more individualized treatment approaches for glioma.

## Materials and methods

### Materials

IDH1 and mIDH1 monoclonal antibodies were purchased from YiKe Company, (ExCell Bio, Shanghai, China). Cell division control protein 2 homolog (CDC2) and bromodomain-containing protein 2 (Brd2) monoclonal antibodies were obtained from Signalway Antibody (College Park, MD, USA), and matrix metalloproteinase-2 (MMP-2) and −9 (MMP-9) monoclonal antibodies and fluorescent labeling goat anti-rabbit IgG (H+L) were purchased from BioWorld Technology, Inc. (Tulare County, CA, USA). High fidelity Platinum *Taq* DNA polymerase, dNTP mix, DNA marker, primers and the DNA Gel Extraction kit were purchased from Beijing Aoke Biotechnology Co., (Beijing, China). Restricted incision enzymes *Bam*HI and *Sac*I, G418 and T4 DNA ligase were purchased from Gibco-BRL (Carlsbad, CA, USA). Lipofectamine 2000 transfection reagent was purchased from Invitrogen Life Technologies (Carlsbad, CA, USA). The U87 human glioma cell line was obtained from the Central Laboratory of Xi’an Jiaotong University (Xi’an, China). U87 cells were propagated in Dulbecco’s modified Eagle’s medium (DMEM), supplemented with 10% fetal bovine serum (FBS) and antibiotics, in a humidified incubator containing 5% CO_2_ at 37°C.

### Construction of the vectors

**Figure 1:**
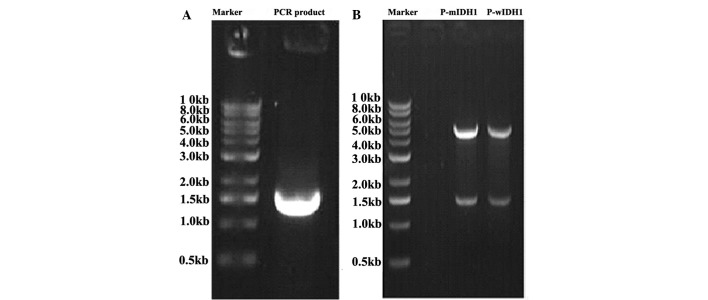
Agarose gel electrophoresis. (A) IDH1 and (B) P-EGFP-wIDH1 and P-EGFP-mIDH1. IDH1, iscocitrate dehydrogenase 1; EGFP, enhanced green fluorescent protein; w, wild-type; m, mutated form.

The IDH1 gene was amplified with the high-fidelity Platinum *Taq DNA* polymerase and detected by 0.8% agarose gel electrophoresis (Fig. 1A). The primers were designed as follows: 5′-ATCGAGCTCA GGAACTGGGGTGATAAGA-3′ (sense primer) and 5′-CGCGGATCCTTCACAAAGGTGGCAATAAC-3′ (anti- sense primer). The final polymerase chain reaction products were cloned into the vector p-enhanced green fluorescent protein gene (EGFP)-C1 and then transferred into DH5α. The recombinant plasmid, named p-EGFP-wIDH1, was treated with the gene site-directed mutagenesis kit following the manufacturer’s instructions and amplified by the same method to obtain p-EGFP-mIDH1. The successful construction of p-EGFP-wIDH1 and p-EGFP-mIDH1 was confirmed by agarose gel electrophoresis (Fig. 1B) and sequence detection.

### Stable transfection and characterization

U87 cells were transfected with vectors p-EGFP-wIDH1, p-EGFP-mIDH1 or p-EGFP-C1 using Lipofectamine 2000 according to the stable transfection procedure. The stably transfected U87 cells were selected by culturing with DMEM containing FBS and 500 μg/ml G418. During the first six days, the FBS content contained in the medium was 10%, whereas seven days following transfection, the content was increased to 15%. The selection lasted for approximately six weeks, until single-cell colonies were formed. Subsequently, the colonies were cultured in DMEM containing 10% FBS and 300 μg/ml G418. The stable cell lines were named U87-EGFP-wIDH1, U87-EGFP-mIDH1 and U87-EGFP.

### Cell morphology observation

Following digestion and culturing of the transfected cells in six-well plates for 24 h under routine conditions, the cells were assayed by fluoroscopy and their morphologies were observed (GPJ9-TS100-F, Nikon, Tokyo, Japan).

### Proliferation by cell counting and Cell Counting Kit (CCK)-8 assay

Cells were adjusted to a density of 2×10^4^ cells/ml and seeded and cultured under routine conditions in six-well plates (4×10^4^ cells per well). Each group of cells was divided into three parallel samples and trypsinized on days 1, 3 and 5 of cell culture. The cell number of each parallel sample was determined by direct cell counting, using a hemocytometer, and the relative growth rate was calculated.

### Cell-cycle distribution analysis

Cells were cultured in 6-well plates for 24 h, harvested and fixed in ice-cold 70% (v/v) ethanol for 24h at 4°C. They were then washed twice with ice-cold phosphate-buffered saline (PBS) and centrifuged at 90 × g for 5 min, followed by treatment with 1 mg/ml RNase for 30 min at 37°C. Following staining with 40 μl propidium iodide (0.1 μg/ml), >10,000 cells per sample were subjected to flow cytometric analysis.

### Flow cytometry for apoptosis determination by the Annexin V-red fluorescent protein (RFP) method

Cells were cultured under routine conditions in a 6-well plate. To determine the number of apoptotic cells, Annexin V-RFP assays were performed using an apoptosis detection kit (Annexin V-RFP Apoptosis Detection kit, Shanghai Ruisai Company, Shanghai, China). At 24 and 48 h time points of the incubation period, cells were harvested and treated according to the manufacturer’s instructions of the Annexin V-RFP kit, and analyzed within 30 min by flow cytometry (BD FACSCanto™ Flow Cytometer, BD Biosciences, New Jersey, NY, USA).

### Transwell assay for migration and invasion ability determination

Cells (2,000 per well) were plated in the upper chamber of a Transwell insert (BD Biosciences, San José, CA, USA) in serum-free DMEM. FBS (10%) served as a chemotactic agent and cells were allowed to migrate for 12 h. The migrated cells were subsequently fixed and stained. Values for migration were obtained by counting the migrated cells under an inverted microscope. Results were expressed as the number of cells identified per random microscope field [mean ± standard deviation (SD)]. To determine the values for invasion, 1×10^4^ cells were seeded in an 8-μm pore polycarbonate membrane chamber inserted in a Transwell insert coated with Matrigel. FBS (15%) served as a chemotactic agent. After 24 h, the migrated cells were fixed, stained and subjected to microscopic inspection. Values for invasion were obtained by cell counting under the inverted microscope.

### Immunofluorescence assay

Cells were seeded on the cover slips in a 24-well plate. After 24 h, cover slips were fixed in 4% paraformaldehyde, rinsed and blocked with 1% bovine serum albumin (BSA). The first antibody (diluted with 1% BSA) was added and incubated overnight at 4°C. Next, cover slips were washed twice with PBS and secondary antibody was added and incubated for 30 min at 37°C. Subsequently, the slides were submitted for fluorescence microscopy observation.

### Western blot analysis

Total protein from the three cell lines was separated electrophoretically in 4–12% SDS-PAGE gels (Ronbio Scientific, Shanghai, China) and transferred to nitrocellulose membranes. Following blocking with 5% non-fat milk for 1 h, the membranes were incubated with the primary antibodies (wild IDH1, mutated IDH1, CDC2, Brd2, MMP-2, MMP-9, β-actin and GAPDH) at 4°C overnight, followed by a 1-h incubation with horseradish peroxidase-conjugated goat-anti-mouse and goat-anti-rabbit secondary antibodies. The membranes were washed thoroughly with Tris-buffered saline containing Tween 20, following each treatment with the antibodies. The bands were visualized by a chemiluminescence method. Data collection and processing were performed using a LAS-3000 luminescent image analyzer (Fujifilm, Tokyo, Japan).

### Statistical analysis

The relative variation rate was statistically analyzed using SPSS 13.0 software (SPSS Inc., Chicago, IL, USA). Data are presented as the mean ± standard deviation. Comparison between groups was performed by one-way analysis of variance. The Student-Newman-Keuls test was used to evaluate the differences between multiple groups. P<0.05 was considered to indicate a statistically significant difference.

## Results

### Stable cell line determination and morphologies of the three stable cell lines

**Figure 2:**
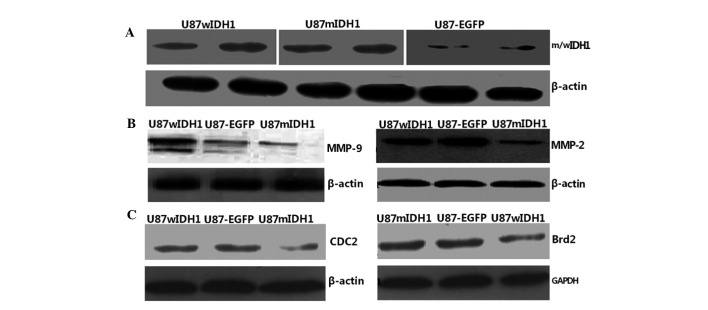
Protein detection by western blotting. Expression of (A) mIDH1 or wIDH1, (B) MMP-2 and −9 and (C) CDC2 and Brd2 in the three cell lines. IDH1, iscocitrate dehydrogenase 1; GFP, green fluorescent protein; w, wild-type; m, mutated form; MMP, matrix metalloproteinase; CDC2, cell division control protein 2 homolog; Brd2, bromodomain-containing protein 2.

Positive GFP signals were observed in the three stable cell lines (U87-EGFP-wIDH1, U87-EGFP-mIDH1 and U87-EGFP), indicating that the target genes had been successfully transfected. Western blotting indicated that the wIDH1 and mIDH1 proteins had been successfully expressed (Fig. 2A). The U87-EGFP-wIDH1 cells exhibited a homogeneous morphology during the primary selection period. These cells characteristically possessed relatively compact, small nuclei and abundant cytoplasm. In addition, the majority of U87-EGFP-wIDH1 cells exhibited a spindle-shaped form, as observed in the original U87 cell line. However, the U87-EGFP-mIDH1 cell line tended to exhibit larger nuclei of various sizes during primary selection and appeared to be more immature. The three cell lines demonstrated no marked differences in morphology when primary selection was complete.

### mIDH1 downregulates the proliferation of U87 cells

**Figure 3:**
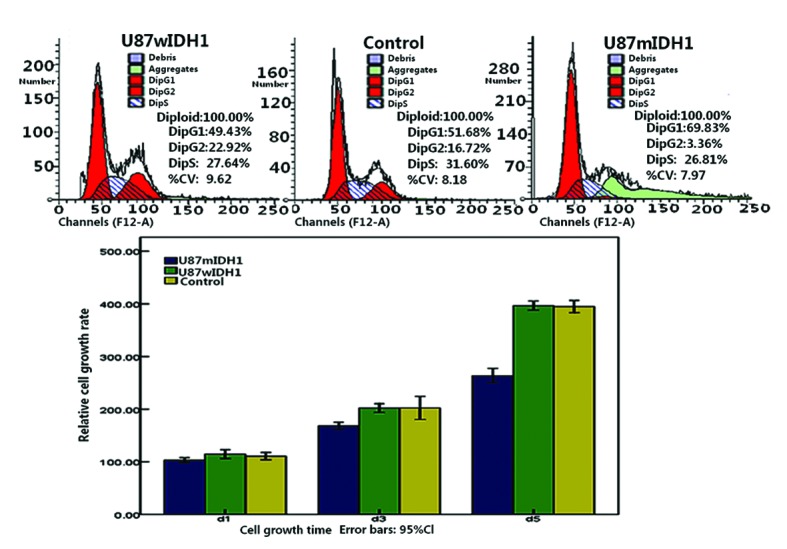
Cell cycle analysis by flow cytometry. (A) U87-EGFP-wIDH1, (B) U87-EGFP and (C) U87-EGFP-mIDH1. (D) Comparison of the relative growth rather of the three cell lines. EGFP, enhanced green fluorescent protein; IDH1, iscocitrate dehydrogenase 1; w, wild-type; m, mutated form.

The relative growth rates of the three cell lines are shown in Fig. 3D. It was found that U87-EGFP-mIDH1 cells had a lower growth rate than the other two groups (P<0.01). There was no significant difference between U87-EGFP-wIDH1 and U87-EGFP cells (P>0.05). The CCK-8 assay exhibited similar results. These results indicate that mIDH1 may function as an inhibitor of glioma cell growth, while wIDH1 did not promote further cell growth.

### mIDH1 alters the cell cycle of the U87 cell line

Cell cycle analysis showed that U87-EGFP-mIDH1 had a higher number of cells in the G1 phase following cell culture for 24 h. The number of U87-EGFP-mIDH1 cells arrested in the G1 phase increased and those in the G2/M stage decreased, compared with U87-EGFP-wIDH1 and U87-EGFP cells. wIDH1 cells demonstrated a non-significant difference in the cell cycle from the U87-EGFP controls (Fig. 3A–C).

### mIDH1 does not affect the apoptosis rates under routine conditions

The Annexin V/RFP method indicated that the apoptosis rates of all the three groups varied by ~1%. There were no significant differences between the three groups under routine culture conditions (P>0.05), indicating that mIDH1 had no effect on the apoptosis rate of glioma cells.

### mIDH1 increases cell migration and reduces the cell invasion

**Figure 4:**
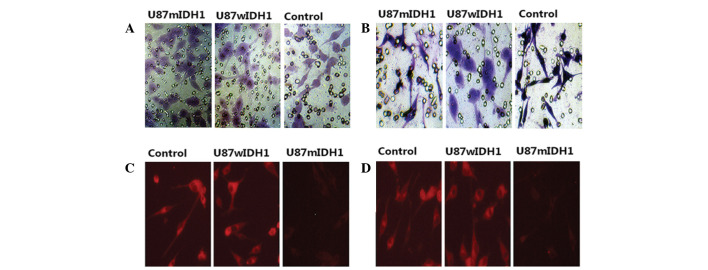
(A) Invasion and (B) migration ability detection by transwell assay and (C) MMP-2 and (D) MMP-9 detection by mmunofluorescence assay in three cell lines. (A) U87mIDH1 had a higher migration ability than U87wIDH1 and the U87-EGFP control. (B) The invasion ability of U87mIDH1 was lower than that of U87wIDH1 and the U87-EGFP control. (C) MMP-2 and (D) MMP-9 expression in U87-EGFP and U87wIDH1 was higher than that of U87mIDH1. (magnifcation, ×200). EGFP, enhanced green fuorescent protein; IDH1, iscocitrate dehydrogenase 1; w, wild-type; m, mutated form; MMP, matrix metalloproteinase.

The migration and invasion abilities of cells were measured by serum chemotaxis. The numbers of U87-EGFP-mIDH1, U87-EGFP-wIDH1 and U87-EGFP cells that migrated into the membranes were ~125.5±8.7, ~78.5±7.4 and ~73.8±8.2 cells per microscopic field (Fig. 4A). The results indicated that the mutated cells had a higher migration ability than the other two groups (P<0.01). No significant difference was detected in the migration rate between U87-EGFP-wIDH1 cells and the control cells (P>0.05). However, IDH1 mutation reduced the invasion ability of glioma cells. A small number of U87-EGFP-mIDH1 cells (28.3±4.6) invaded the membrane, compared with the other two groups of cells (P<0.01; Fig. 4B). Among the three groups, the morphology of the U87-EGFP-mIDH1 cells changed the most, following invasion of the membrane. U87-EGFP-wIDH1 cells (65.4±6.8) demonstrated little difference in invasion ability compared with the control cells (68.2±7.6) (P>0.05).

### mIDH1 induces downregulation of CDC2 levels and upregulation of Brd2 levels

Western blotting and gray-scale scanning analysis revealed that U87-EGFP-mIDH1 expressed relatively low CDC2 levels compared with the other two cell lines (P<0.01; Fig. 2C). No significant difference was found in the CDC2 levels between the U87-EGFP-wIDH1 and U87-EGFP cells. U87-EGFP-mIDH1 exhibited higher Brd2 protein expression than the other two cell lines (P<0.01).

### mIDH1 reduces downregulation of MMP-2 and MMP-9 levels

Immunofluorescence (Fig. 4C and D) and western blotting (Fig. 2B) demonstrated that U87-EGFP-mIDH1 exhibited low expression levels of MMP-2 (Fig. 4C) and MMP-9 (Fig. 4D) when compared with the other two cell lines.

## Discussion

IDH has an important role in cell metabolism. Though different IDH enzymes may have independent functions, they are all related to the NADPH pool of cells according to the activities of lipogenesis, antioxidation and immune system response (11,12). IDH function disorders may cause changes to the NADPH pool and lead to cell metabolism disorders (10).

IDH1 mutation in glioma has attracted much attention. Mutation may alter the normal cell metabolism mechanisms and prevent complete conversion of isocitrate to α-ketoglutarate. In addition, mutation has been shown to cause 2-hydroxyglutarate and hypoxia inducible factor-1α accumulation, leading to the added risk of tumorigenesis (13). This characteristic may be attributed to oncogene, but this has not been widely validated. Furthermore, these observations do not correspond with the high incidence seen with specific congenital and metabolic disorders. A recent study suggested that the metabolic levels of 2-hydroxyglutarate were not associated with tumorigenesis or tumor malignancy (14). Therefore, further studies are needed to elucidate the specific role of IDH1 mutation in tumorigenesis. Due to IDH1 mutation, glioma is associated with two subtypes which bring about distinctly different prognoses (7,15,16). An increasing number of studies have revealed that IDH1 mutation carriers have an improved prognosis, meaning that IDH1 mutations may confer a protective effect. A study by Zhu *et al* was consistent with these observations (17). The present study focused on the biological changes induced by IDH1 mutation in glioma cells. The mechanisms underlying the effect of IDH1 mutation on the enhanced prognosis of glioma patients was also investigated, in the hope of providing clues to improve glioma therapy.

Cell cycle control is regulated by checkpoint control via cell cycle regulatory proteins, including cyclins and cyclin-dependent kinases. This process is an important means of inhibiting cancer cell growth and division (18). CDC2 is a fission yeast CDC2 gene. Cells are unable to enter mitosis without CDC2 activity, which implies that CDC2 is a key regulator of fission yeast mitosis. A number of drugs have been designed as antitumor drugs for targeting CDC2 (19). Qiao *et al* previously reported that DAT-230 reduces CDC2 levels and induces G2/M phase arrest and apoptosis in tumor cells (20). Cell cycle analysis in the present study showed that IDH1 mutation led to reduced CDC2 levels and caused increased G1 phase length and reduced S and G2 phases. Downregulated CDC2 levels may lead to phosphorylation deficiencies of histone H1 and nuclear lamins, hinder mitotic spindle formation and further inhibit the cell cycle. CDC2 downregulation markedly reduced cell proliferation in the present study, which is consistent with a study by Hogan *et al* (21). U87-EGFP-wIDH1 and U87-EGFP cells exhibited a relatively normal distribution within the cell cycle stages. Compared with U87-EGFP, U87-wIDH1 had a slightly increased G2 phase, which is likely to be due to the different patterns of energy metabolism between the two cell lines. This result implies that the IDH1 gene may play a complementary role in cellular mitosis.

Cell cycle regulation is a complex process involving multiple factors. Brd2 belongs to the bromo and extra terminal (BET) protein family and has been implicated in fundamental cellular processes, including cell cycle and transcriptional regulation. Brd2 is closely associated with the cell cycle transcription factors, E2F1 and E2F2 (22). Ottinger *et al* have hypothesized that a BET protein, in combination with the MHV-68 orf73 protein, activates the promoters of G1/S cyclins, while gene mutation in one binding site inhibits the interaction between Brd2 and E2F and reduces the responses of the BET protein to the promoters of cyclin D1, D2 and E (23). Ectopic expression of Brd2 has been found to inhibit S phase progression and induce G1 cell cycle arrest or exit and Brd2 knockdown promotes S phase entry (24). These observations are consistent with findings of the present study. In this study, IDHI mutation resulted in an increased proportion of cells in the G1 phase and increased Brd2 levels in glioma cells. A previous study has shown that Brd2-deficient embryonic fibroblast cells proliferate more slowly than those of wild-type fibroblast cells (25). The present study demonstrated that glioma cells with mutated IDH1 had higher Brd2 levels and proliferated more slowly than the other two groups. The U87-mIDH1 cells exhibited a lower proliferation rate than the U87-wIDH1 cells, which is likely to be due to blockage of the U87-mIDH1 cell cycle. From this, it is possible to conclude that IDH1 mutation plays an inhibitory role in cell proliferation, due, in part, to blockage of the cell cycle. This may partly explain why glioma patients with mutated IDH1 have a longer survival period. Although IDH1 mutation may not be used as an independent factor for improved prognosis, it is likely to block the cell cycle and to inhibit cell proliferation.

MMPs, a family of proteolytic enzymes, help increase the invasion potential of tumor cells by remodeling the extracellular matrix. MMP expression has been found to be associated with tumor progression and survival in several types of human cancers. In the present study, cells with mutated IDH1 exhibited low expression of MMP-2 and MMP-9 and low invasion ability, compared with the other two groups. This was shown by a Transwell invasion experiment. From this perspective, mutated IDH1 cannot be regarded simply as an oncogene. As glioma patients with mutated IDH1 have a low capacity for degrading various components of the extracellular matrix, surgery may result in increased opportunity for invasion of glioma cells. This may partly explain why the pathological diagnoses of specific patients are promoted to a higher grade following the initial operation. However, in the present study, IDH1 mutation induced changes in the biological behavior of glioma cells and in the mechanism of tumorigenesis. IDH1 mutation downregulated glioma cell proliferation by blocking the cell cycle, reduced the cell invasion ability via MMP-2 and MMP-9 downregulation and promoted the cell migration ability. There is reason to believe that glioma patients with mutated IDH1 may have a better prognosis due to blockage of the cell cycle and inhibition of cell invasion ability.

However, IDH1 mutation cannot be regarded as exclusively responsible for this enhanced prognosis. The apoptosis rate did not increase accordingly in the present study; although, when treated by chemotherapy and radiotherapy, the result may be different. This is since normal IDH1 enzymes contribute significantly to the NADPH levels and provide considerable protection against oxidative stress from chemotherapy and radiation, which is of great importance in ensuring normal IDH1 regulation. Evidence of this has been identified in several other tumors (26). Mutated IDH1 may fail to provide more energy to repair the oxidative stress injury from chemotherapy and radiation. Mateescu *et al* (27) indicated that although oxidative stress promotes tumor growth, it also sensitizes tumors to treatment.

In conclusion, the present study demonstrated that IDH1 mutation blocks the cell cycle and inhibits cell proliferation by downregulating CDC2 levels and increasing Brd2 levels. IDH1 mutation was markedly associated with a significantly reduced invasion ability, by reducing the levels of MMP-2 and MMP-9. This study is expected to provide a partial explanation for the improved prognosis of patients with mutated IDH1 genes. It may be concluded that IDH1 mutation improves the prognosis of glioma patients by altering the cell cycle, inhibiting cell proliferation and downregulating cell invasion ability.
